# Portal vein thrombosis following targeted-release budesonide in IgA nephropathy and occult liver disease

**DOI:** 10.1093/ckj/sfag372

**Published:** 2026-10-22

**Authors:** Gerald S Braun, Claudia Seikrit

**Affiliations:** Department of Nephrology and Rheumatology, RWTH University Hospital, Aachen, Germany; Department of Nephrology, Sana Klinikum Oberfranken, Coburg, Germany; KfH Nierenzentrum, Coburg, Germany; School of Medicine, University of Split, Split, Croatia; Department of Nephrology and Rheumatology, RWTH University Hospital, Aachen, Germany

**Keywords:** budesonide, first-pass metabolism, hepatic dysfunction, hepatocellular carcinoma adverse drug-reaction, hereditary hemochromatosis, IgA nephropathy, Nefecon, portal vein thrombosis

## Abstract

Targeted-release budesonide (Nefecon) is the first gut–kidney axis-directed therapy for high-risk immunoglobulin A nephropathy (IgAN) and relies on intact hepatic first-pass metabolism to limit systemic exposure. We report a 63-year-old man who developed acute portal vein thrombosis within 3 days of treatment initiation. Imaging demonstrated near-complete thrombosis of the portal venous system. Subsequent evaluation revealed previously unrecognized chronic liver disease, including hereditary hemochromatosis, alcohol use, and early hepatocellular carcinoma without vascular invasion. In conjunction with three previously reported cases of splanchnic vein thrombosis in patients receiving budesonide to treat advanced autoimmune hepatobiliary disease and published pharmacokinetic data, this observation suggests that hepatic dysfunction and disease may lower the thrombotic threshold for budesonide. These findings support careful assessment of hepatic function when initiating targeted-release budesonide in IgAN.

## INTRODUCTION

Immunoglobulin A nephropathy (IgAN) is the most common glomerular disease worldwide and may progress to kidney failure in up to 50% of patients [1]. Current guidelines emphasize optimized supportive care, with additional therapy for patients at high risk of progression [2]. Targeted-release-budesonide (Nefecon; also marketed as Tarpeyo^®^, Kinpeygo^®^, or Nefecon^®^) represents the first approved disease-specific immunomodulatory therapy for such patients [3]. By delivering glucocorticoid activity to gut-associated lymphoid tissue while undergoing extensive first-pass hepatic metabolism, the formulation is designed to maximize local efficacy while limiting systemic exposure [4]. This therapeutic concept, however, inherently depends on preserved hepatic metabolic capacity. Accordingly, regulatory labeling contraindicates its use in severe hepatic impairment (Child–Pugh class C) and recommends clinical monitoring for signs of increased systemic steroid actions and adverse events in moderate liver impairment (Child–Pugh class B) [5, 6].

Glucocorticoids, including budesonide, are associated with a dose-dependent risk of venous thromboembolism mediated by effects on platelets, coagulation pathways, and endothelial function [7]. Recent observations of a total of six cases suggest that pulmonary thromboembolic complications may occur with both conventional and targeted-release budesonide for IgAN, even in the absence of overt risk factors [8, 9] (Braun et al., *Clin Kidney J*, in press; *N* = 4 cases; abstract and graphical abstract provided as Supplementary material for reviewer reference). Earlier reports in hepatobiliary budesonide indications described rare cases of splanchnic vein thrombosis—including portal vein thrombosis and Budd–Chiari syndrome—in patients with advanced liver disease treated with oral budesonide, pointing to a potential interaction between impaired hepatic function and thrombotic risk (see below).

Against this background, here we report a case of acute portal vein thrombosis occurring shortly after initiation of targeted-release budesonide for IgAN in a patient in whom pre-existing chronic liver disease had not been recognized as a potential risk factor prior to treatment initiation, and which became apparent only after thorough evaluation following the event. In the context of prior published observations and the pharmacologic properties of budesonide, this case raises the possibility that clinically inapparent liver disease may represent underrecognized determinants of risk when initiating targeted-release budesonide in IgAN.

## CASE REPORT

Complementary details of the present case and three previously reported cases in which budesonide treatment for primary biliary cholangitis (PBC) or autoimmune hepatitis (AIH) was followed by portal or hepatic vein thrombosis are provided in Table 1. Imaging findings and the laboratory time course of the present case are further summarized in Fig. 1 and Table S1. A 63-year-old Caucasian man presented with new-onset nephrotic syndrome and hematuria. His medical history included arterial hypertension and unexplained arterial thrombosis of the arm 14 years earlier, successfully treated by endovascular thrombectomy. Medications included amlodipine 10 mg, bisoprolol 5 mg, torasemide 20 mg, allopurinol 300 mg, and aspirin 100 mg daily. He smoked and reported regular alcohol consumption, raising suspicion of chronic alcohol use, although its extent remained unclear. He worked as a plumber servicing industrial furnaces, frequently in confined spaces and at high ambient temperatures, with several consecutive days of out-of-town work. He was married with an adult daughter.

**Figure 1:**
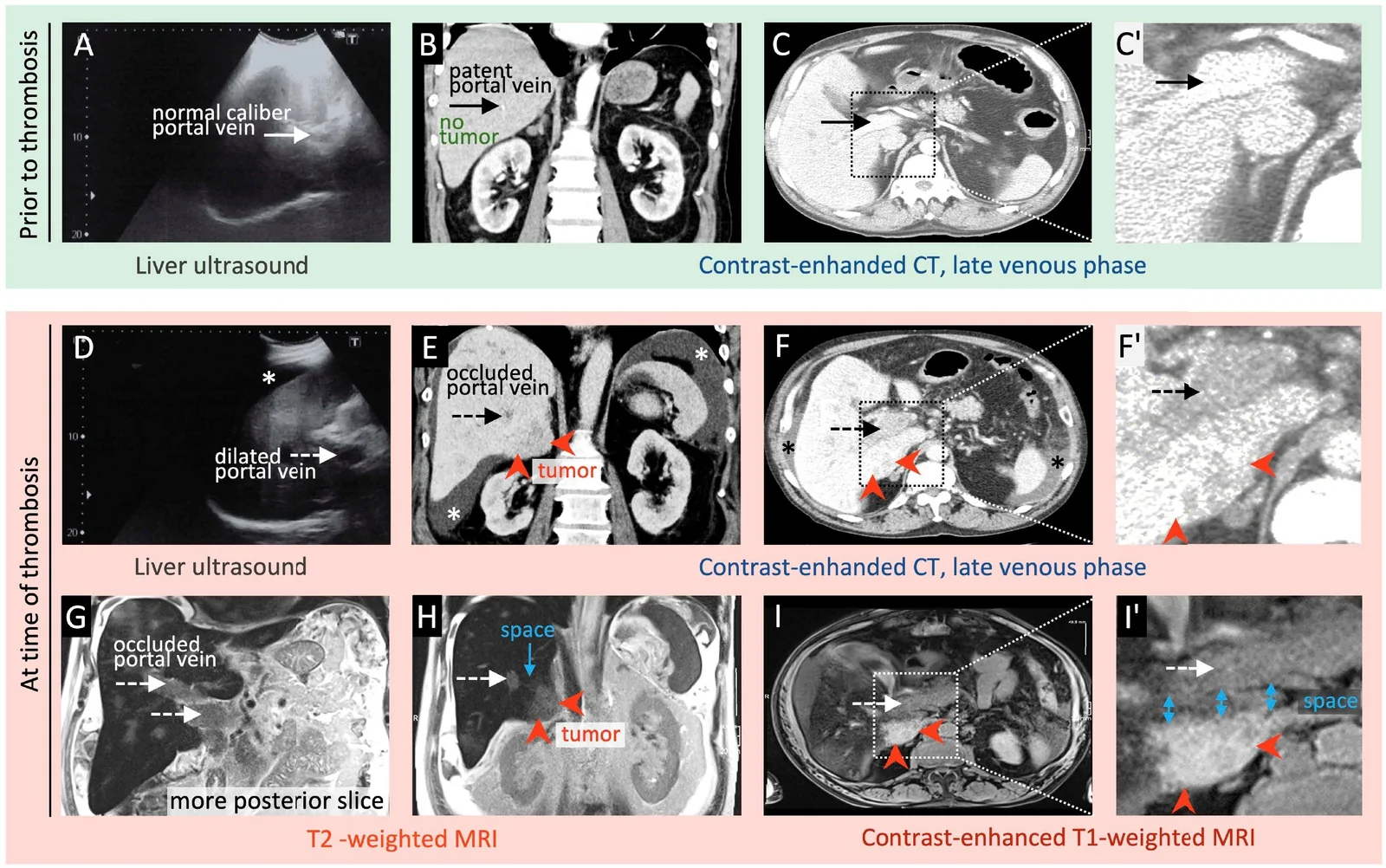
Imaging findings before and at the time of portal vein thrombosis. (A–C′) Normal imaging findings prior to the thrombotic event. Liver ultrasound and contrast-enhanced CT obtained 4 months and 15 years earlier, respectively, demonstrate a portal vein of normal caliber with preserved contrast perfusion (solid arrows). (D–I′) Pathologic findings at the time of thrombosis. Imaging shows a dilated, non-perfused (occluded) portal vein (dashed arrows). Asterisks denote ascites. Red arrowheads indicate a newly identified hepatic lesion consistent with HCC. Blue arrows denote preserved liver parenchyma between the tumor and the portal vein, supporting the absence of vascular invasion. Imaging methodology: (A, C–D, F, F′, H, I′) transverse planes; (B, E, G, H) coronal planes. Ultrasound was performed using a Toshiba Xario system. Contrast-enhanced CT before thrombosis was performed on a 16-slice scanner (120 kV, 47 mAs) and CT at the time of thrombosis on an 80-slice scanner (100 kV, 138 mAs). MRI was performed on a 1.5 T system.

**Table 1:** Present and literature cases of budesonide-associated hepatic region thrombosis.

Case #/publication	Age (years), sex	Dx	Site of thrombosis	Formulation	Time to event (days)	Key diagnostic and clinical features
Case 1—this report	63, M	IgAN	Portal vein	Nefecon (Kinpeygo^®^) 16 mg once daily	3	Electron microscopy not suggestive for minimal change disease. Nephroprotective therapy initiated at diagnosis: renin–angiotensin system inhibitor, sodium–glucose co-transporter 2 inhibitor, mineralocorticoid receptor antagonist. Unrecognized liver disease (alcohol use disorder, homozygous HFE C282Y mutation with hepatocellular carcinoma, liver cirrhosis Child–Pugh A); further hepatologic workup: isolated LC1 positivity (interpreted as secondary phenomenon); other tests negative/normal: p-ANCA, c-ANCA, ANA, AMA, SLA, alpha-1 antitrypsin, ceruloplasmin, viral hepatitis A, B, C, and E. Mild mixed cryoglobulinemia type II/III, negative immunofixation, serum IgA mildly elevated. Thrombophilia testing negative (LA, aCL, anti-β2GPI, FVL, APCR, protein S deficiency, F2 G20210A, AT deficiency). History of idiopathic brachial arterial thromboembolism 14 years earlier. Daughter with venous thrombosis
Case 2 Hempfling 2003 [10]	60, F	PBC	Portal vein	Budenofalk^®^ 9 mg once daily	10	Budesonide PBC pilot study; treatment discontinued on day 10 due to weight gain; portal vein thrombosis diagnosed 12 days later; no further details available
Case 3 Hempfling 2003 [10]	57, F	PBC	Portal vein	Budenofalk^®^ 9 mg once daily	50	Budesonide PBC pilot study; portal vein thrombosis diagnosed ∼1 month after treatment completion; death within 6 weeks
Case 4 Mederacke 2012 [11]	40, F	AIH	Hepatic veins (Budd–Chiari syndrome)	Budenofalk^®^ 3 mg three times daily	28	Long-standing AIH with acute flare; budesonide used as step-down therapy from prednisolone; hepatocellular carcinoma diagnosed 2 months after the thrombotic event; thrombophilia testing negative (JAK2 V617F, FVL, APCR)

Note: Approximate dates are indicated where exact dates were unavailable.

Abbreviations: aβ2GPI, anti-β2-glycoprotein I antibodies; aCL, anticardiolipin antibodies; AIH, autoimmune hepatitis; AMA, anti-mitochondrial antibody; ANA, antinuclear antibody; ANCA, antineutrophil cytoplasmic antibody; APCR, activated protein C resistance; AT, antithrombin; F2 G20210A, prothrombin G20210A mutation; FVL, factor V Leiden; HFE, homeostatic iron regulator; IgAN, immunoglobulin A nephropathy; JAK2 V617F, Janus kinase 2 V617F mutation; LA, lupus anticoagulant; LC1, anti-liver cytosol type 1 antibody; PBC, primary biliary cholangitis; SLA, anti-soluble liver antigen antibody.

On presentation, he weighed 104 kg (height 176 cm) with a blood pressure of 122/86 mmHg. Examination revealed pronounced bilateral pitting edema with chronic skin changes and two healing traumatic ulcerations (3–5 cm) on the left foot and shin, attributed to congestion; arterial Doppler ultrasonography showed no relevant perfusion deficits.

Abdominal ultrasonography showed normal-sized kidneys (11.2 and 12.2 cm) with mildly reduced parenchymal thickness and an enlarged, homogeneously steatotic liver without overt signs of cirrhosis. There was no ascites or urinary tract obstruction. Echocardiography was unremarkable (left ventricular ejection fraction 65%).

Laboratory findings included an eGFR of 55–57 ml/min/1.73 m² by creatinine and cystatin C, proteinuria of 7.41 g/day (albuminuria 5.99 g/day), albumin 28 g/l, IgA 527 mg/dl, immunoglobulin G 896 mg/dl, and low-density lipoproteincholesterol 158 mg/dl; hepatitis B, hepatitis C, and HIV serologies were negative. Transaminases and γ-glutamyl transferase were mildly elevated (≤2 × upper limit of normal), without raising suspicion of advanced liver disease. Transferrin saturation was 93.5% despite normal serum iron (32.9 µmol/l) but was not recognized as indicative of clinically relevant iron overload.

Kidney biopsy established IgAN (MEST-C M1E1S0T0-C1). Of 13 glomeruli, four showed endocapillary and two extracapillary proliferation. In the absence of chronic inflammatory disease, and given the uncertain extent of alcohol consumption, no clinically overt advanced liver disease in our assessment, negative antineutrophil cytoplasmic antibody and antinuclear antibody, normal immunofixation, and only non-specific liver abnormalities, there was insufficient evidence at the time to suggest secondary IgAN. Given stable kidney function, with eGFR initially increasing transiently to ∼70 ml/min/1.73 m² during diuretic treatment, fragile skin, concerns regarding medication adherence during frequent work-related travel, and the patient’s reluctance toward systemic immunosuppression, supportive treatment was chosen. He was discharged on ramipril 5 mg and atorvastatin 10 mg daily; ramipril was subsequently increased to 10 mg and dapagliflozin 10 mg added.

At 5.3 months, urinary albumin-to-creatinine ratio (UACR) had decreased to 2.73 g/g and the clinical nephrotic syndrome had resolved. Consequently, targeted-release budesonide (Nefecon) was recommended to provide IgAN-specific treatment while limiting systemic glucocorticoid exposure. Due to delayed patient implementation, treatment was initiated only at 6.4 months from the initial presentation.

Within 3 days, the patient developed severe abdominal pain, distension, and peripheral edema. Imaging revealed portal vein thrombosis, and anticoagulation was initiated; antibiotics were administered for suspected secondary peritonitis. Nefecon, discontinued by the patient at symptom onset, was withheld. Thrombophilia testing was negative (see Table 1 for details). An acute event was supported by new portal vein dilatation and ascites on ultrasound and contrast-enhanced computed tomography (CT) (Fig. 1D-F′), which were absent on prior imaging, including ultrasound 4 months earlier (Fig. 1A) and CT obtained 15 years before during a previous medical workup (Fig. 1B–C′). Near-complete thrombosis of the portal venous system extending into peripheral intrahepatic branches [∼90% liver involvement; best seen on magnetic resonance imaging (MRI), Fig. 1G], together with a transient 1.5- to 3-fold increase in bilirubin, transaminases, cholestatic parameters, and lactate dehydrogenase further indicates an acute onset (see Table S1 for laboratory course).

Imaging also revealed previously undetected hepatic masses (at the junction of segments I and VI: 5.3 cm × 3.9 cm; segment VIII: 2.6 cm), consistent with hepatocellular carcinoma (HCC) based on morphology (Fig. 1E–F′). There was no evidence of tumor invasion into the portal venous system, excluding a direct thrombotic effect from local vascular obstruction (Fig. 1H–I′). Subtle morphologic features of cirrhosis were present, including a mildly nodular hepatic contour and caudate lobe hypertrophy.

Further hepatologic workup supported HCC, with markedly elevated alpha-fetoprotein (∼1600 ng/ml), increasing more than 25-fold over the subsequent 6 months. As a key etiologic finding, the workup also established hereditary hemochromatosis, with persistently elevated transferrin saturation (∼85%–90%), markedly elevated ferritin (∼2500 µg/l), and homozygosity for the HFE C282Y mutation.

When confronted with the hepatologic findings, the patient disclosed higher alcohol consumption than previously reported. In retrospect, the skin changes contributing to our reluctance toward systemic glucocorticoids may partly have reflected hemochromatosis. His previous brachial artery thrombosis and his daughter’s unprovoked pulmonary embolism despite negative thrombophilia screening raised the possibility of a non-classical familial thrombotic predisposition, potentially related to hemochromatosis. Hemochromatosis screening of his daughter was recommended, but results were unavailable due to loss to follow-up. Six weeks after the thrombotic event, treatment with lenvatinib was initiated for HCC, and anticoagulation was maintained throughout. The patient died of tumor progression 7 months after the thrombotic event.

## DISCUSSION

Budesonide-associated splanchnic vein thrombosis was first reported two decades ago in patients treated for autoinflammatory hepatobiliary diseases who developed portal vein thrombosis and Budd–Chiari syndrome, respectively [10, 11]. Two cases arose from a pilot study in PBC [10], and one from a patient with AIH treated for an acute flare [11] (summarized as cases 2–4 in Table 1). Together with hemochromatosis, these conditions share a pathophysiological pattern of declining hepatic function and chronic low-grade inflammation, promoting fibrosis, cirrhosis, and ultimately HCC [12–15]. The PBC cases involved advanced cirrhosis (stages III–IV; approximately Child–Pugh B/C), and the AIH case—similar to ours—was incidentally diagnosed with developing HCC in temporal association with thrombosis (Table 1). Across all four cases, thrombosis occurred rapidly after budesonide initiation (within 3–50 days, see Table 1). In contrast, among previously reported IgAN cases without liver disease in which pulmonary embolism developed after initiation of budesonide, time-to-event data were available in five cases and were substantially longer (mean 209 days, range 60–360), while one case lacked such data. The earliest event (60 days) occurred in a patient with an additional risk factor (hyperthyroidism) [8] (Braun et al. *Clin Kidney J*, in press). This kinetic pattern suggests a strong modifying effect of liver disease on thrombotic risk under budesonide and warrants evaluation in the context of the drug’s pharmacokinetics and hepatic metabolism.

Budesonide, originally developed as a locally acting therapy for respiratory disease, was later adapted for gastrointestinal use with multiple enteric-coated formulations [16, 17]; in IgAN, it is applied as a targeted-release formulation acting on the gut–kidney axis in the terminal ileum [18]. A synoptic overview of formulations is provided on the left-hand portion of Table 2.

**Table 2:** Comparative characteristics and pharmacokinetics of budesonide formulations.

Formulation	Formulation technology and release characteristics ^a^	Predominant site of drug release—approved indications	Study (author, year)	Dosing Regimen	Study purpose	*C*_max_ (ng/ml)	AUC_∞_ (ng h/ml)	*T*_max_ (h)	*T*_1/2_ (h)
Rectal enema	Not applicable	Rectum/sigmoid colon—ulcerative colitis	Danielsson, 1987 [24]	Single 2 mg dose (×4 = 8 mg)	Quantify systemic absorption after rectal application	0.9; (∼3.6)^b^	4.2; (∼16.8)^b^	1.3	N/A
Cortiment^®^ (Uceris^®^)	Multi-matrix-system (MMX)—pH trigger ≥7.0; 0% (0–180 min)	Colon—ulcerative colitis	Brunner, 2006 [25]	Single 9 mg dose	Characterize MMX (pH > 7.0) full-colon release profile	1.4	14.8	∼9.8	N/A
Entocort^®^	Enteric-coated controlled ileal release (EC/CR)—pH trigger ≥5.5; 70% (0–60 min), 15% (60–120 min)	Ileum/ascending colon—Crohn’s disease (ileocecal)	Edsbäcker, 2004 [20]	Single 9 mg dose	Establish baseline PK profile in healthy adults	1.8	13.6	4.0	2.8
			Barratt, 2024 [26]	Single 9 mg dose	Comparator for the Nefecon (Nef-101 study)	1.76	12.2^c^; (∼13.5)^c^	3.38	4.95
Budenofalk^®^	Enteric-coated granules/pellets—pH trigger ≥6.4; 0% (0–120 min), 50% (120–180 min)	Terminal ileum/ascending colon—Crohn’s disease; autoimmune hepatitis; microscopic colitis	Miehlke, 2018 [27]	Single 9 mg dose	Characterize pH-pellet (>6.4) release profile in healthy adults	∼3.6	∼17.9^c^	∼5.4	∼4.0
			Hempfling, 2003 [10]	Single 3 mg dose (×3 = 9 mg)^d^	Assess PK profile in patients with early-stage liver disease (PBC) I/II	1.51; (∼4.53)^d^	5.14; (∼15.42)^d^	4.8	∼2.3^e^
				Single 3 mg dose (×3 = 9 mg)^d^	Assess PK impact of severe liver failure (cirrhosis) IV	4.90; (∼14.70)^d^	23.15; (∼69.45)^d^	5.0	∼5.8^e^
Nefecon (Tarpeyo^®^, Kinpeygo^®^, Nefegan^®^)	Enteric-coated targeted release (Targit^®^)—pH trigger ∼6.0–6.5; 5% (0–60 min), 85% (60–120 min)	Distal ileum—IgAN (high risk of progression)	Barratt, 2024 [26]	Single 8 mg dose	Characterize PK profile of a novel targeted-release formulation (Nef-101 study)	3.33	14.1^c^; (∼15.0)^c^	3.80	4.27
				Single 16 mg dose	Assess dose proportionality of the Nefecon formulation (Nef-101 study)	6.26	27.1^c^; (∼31.7)^c^	3.96	4.40

Note: *T*_max_ and *t*_1/2_ are rate parameters and are dose-independent. PK parameters derived from single-dose studies in adults unless otherwise specified.

Abbreviations: AUC_∞_, area under the concentration–time curve; *C*_max_, maximum plasma concentration; *t*_1/2_, elimination half-life; *T*_max_, time to maximum plasma concentration.

a Derived from *in vitro* dissolution data simulating gastrointestinal transit (USP < 711>, two-stage method B; pH 1.2 → 6.8); not directly comparable to *in vivo* exposure. Data adapted from reference [28].

b Linear extrapolation to an equivalent 8 mg dose.

c AUC values represent AUC₀_–_₂₄ when AUC_∞_ was not reported; values in parentheses indicate estimated AUC_∞_. AUC₀_–_₂₄ approximates AUC_∞_ (+7% Entocort^®^, +11% Nefecon; Nef-105).

d Linear extrapolation to an equivalent 9 mg dose.

e *t*_1/2_ values from Hempfling *et al*. [10] are reported as medians.

Chemically, budesonide is a potent non-halogenated glucocorticosteroid composed of the 22R and 22S epimers of 16α-hydroxyprednisolone, with ∼15-fold higher glucocorticoid receptor affinity than prednisolone and ∼200-fold greater anti-inflammatory potency than hydrocortisone [19, 20]. Extensive first-pass metabolism limits systemic exposure (oral bioavailability 9%–21%, plasma clearance ∼70–85 l/h, half-life ∼3 h) [20–22]. While native budesonide is not excreted via the kidneys, its major metabolites, which exhibit <1% activity of the parent compound, are largely excreted in urine [5, 6].

The above pharmacokinetics are largely determined by CYP3A4-mediated metabolism. CYP3A4 inhibitors, such as ketoconazole or grapefruit juice, may increase budesonide exposure by ∼6.5–8- and ∼2-fold, respectively, whereas inducers decrease exposure [5, 6] (see Braun et al. *Clin Kidney J* for a comprehensive list of interacting agents). Liver disease represents another key condition associated with reduced CYP3A4 activity, resulting in increased systemic exposure, as discussed below [20].

Differences between budesonide formulations further modify systemic exposure. Nefecon is administered at 16 mg/day compared with 9 mg/day for conventional gastroenterological formulations. While comparable single doses yield similar areas under the curve (AUC), reflecting cumulative systemic exposure, Nefecon produces approximately twice the peak plasma concentration (*C*_max_). At standard dosing, this translates into an approximately four-fold higher peak concentration compared with conventional budesonide used in inflammatory bowel disease (Table 2). These differences reflect distinct release kinetics and absorption profiles, as summarized including corresponding references in Table 2. The prescription information for Nefecon reports pharmacokinetic parameters consistent with these findings (single 16 mg dose: *C*_max_ 4.4 ng/ml; AUC_0–24_ 24.1 h ng/ml; time to maximum plasma concentration [*T*_max_] 5.1 h; elimination half-life [*t*_1/2_] 5.0–6.8 h) [5, 6].

In hepatic impairment, these effects are amplified. Reduced hepatocyte mass and portosystemic shunting of up to 75% of hepatic blood flow markedly diminish first-pass metabolism, increasing systemic exposure and prolonging half-life [23]. The aforementioned pilot study of budesonide in PBC, [10] which reported cases 2 and 3 as adverse events (cases 2 and 3 in Table 1), also included pharmacokinetic analyses demonstrating a 4.5-fold higher systemic exposure in advanced versus early-stage disease (Table 2). Notably, the two individuals who subsequently developed portal vein thrombosis exhibited markedly impaired clearance (14–21 ml/min/kg vs 156 ml/min/kg in early-stage disease) and more advanced portal hypertension [10]. Consistent findings are reflected in the prescribing information for Nefecon, reporting a 3.5-fold increase in bioavailability in Child–Pugh class B hepatic impairment (27% vs 7.4% in healthy individuals) and a 1.4-fold increase in Child–Pugh class A [5, 6].

Severe hepatic inflammation exerts a similar effect. Inflammatory suppression of CYP3A4 activity has been associated with up to 13-fold higher serum budesonide levels and 2–4-fold reductions in metabolite-to-parent ratios, indicating impaired metabolic conversion despite preserved shunt fractions (∼5%) [23].

## CONCLUSION

We report a case of portal vein thrombosis occurring rapidly after initiation of targeted-release budesonide for IgAN in a patient with previously unrecognized severe liver disease. Our case parallels previous reports in which conventional budesonide has been implicated as a trigger of portal or hepatic vein thrombosis in the presence of risk factors. Impaired hepatic first-pass metabolism, a proinflammatory milieu, and genetic susceptibility may converge to lower the thrombosis threshold. Targeted-release budesonide (Nefecon) is administered at higher doses than conventional budesonide (16 mg vs. 9 mg) and results in greater systemic exposure, which may increase further with impaired hepatic metabolism. Thus, budesonide may act as an iatrogenic tipping point for thrombosis in susceptible individuals, with safety dependent on hepatic metabolic capacity.

We suggest a history indicative of hepatic dysfunction be carefully considered before initiating Nefecon, even in the absence of overt cirrhosis. Clinical or laboratory clues, such as skin changes or findings suggestive of iron overload or other hepatic disease, should, in our view, prompt targeted investigation. However, given the rarity of this observation, our case does not justify extensive routine hepatologic screening of all patients before Nefecon initiation. Paradoxically, Nefecon was chosen partly to limit systemic glucocorticoid exposure in a medication-reluctant patient with fragile skin and ulcerations; in retrospect, this seemingly cautious approach may not have proved beneficial.

Current prescribing information for Nefecon only addresses Child–Pugh classes B and C, recommending monitoring in class B and avoidance in class C. Further studies are needed to better define this potential safety signal.

## Supplementary Material

sfag372_Supplemental_File

## Data Availability

De-identified individual participant data underlying the results reported in this manuscript will be made available upon reasonable request to the corresponding author.
