# Supplementary material for: Portal vein thrombosis following targeted-release budesonide in IgA nephropathy and occult liver disease
Source: Clin Kidney J. 2026 Oct 22;19(11):sfag372. doi: 10.1093/ckj/sfag372 (PMC13760213; doi:10.1093/ckj/sfag372)
Supplement: Supplementary file 1 [file sfag372_supplemental_file.docx]

Braun and Seikrit. Portal vein thrombosis following targeted-release budesonide in IgA nephropathy with occult liver disease

**Supplementary Table 1: Time course of pertinent routine laboratory parameters of case 1**

| **Day** | **1** | **101** | **160** | **194** | **202** | **269** | **381** | **408** |
| --- | --- | --- | --- | --- | --- | --- | --- | --- |
| **Month** | **0** | **3.3** | **5.2** | **6.3** | **6.6** | **8.8** | **12.5** | **13.4** |
| **Clinical course** | **Nephrotic syndrome** | **3-month follow-up** | **5-month follow-up** | **Nefecon initiation**  **(3 days)** | **Admission for portal vein thrombosis** | **2 months post-thrombosis** | **6 months post-thrombosis** | **7 months post-throm-bosis (death)** |
| **Laboratory parameters [reference range]** | | | | | | | | |
| **Hematology** | | | | | | | | |
| Hb (g/dL) [13.5–17.5] | 16.7 | 17.2 | 15.5 | - | 14.2 | 14.9 | 14 | 13.8 |
| MCV (fL) [80–96] | 99 | 101 | 98 | - | 95 | 102 | 106 | 103 |
| Leukocytes (/nL) [4.0–10.0] | 9.9 | 10.4 | 9.3 | - | 17.9 | 11.4 | 10.0 | 11.0 |
| Platelets (/nL) [150–400] | 253 | 334 | 306 | - | 299 | 354 | 318 | 321 |
| **Kidney function** | | | | | | | | |
| eGFR CKD-Epi (mL/min/1.73m^2^) | 55 | 61 | 55 | - | 47 | 27 | 25 | 5 |
| eGFR Cystatin C (mL/min/1.73m^2^) | 57 | 43 | - | - | - | - | 20 | - |
| UPE (mg/day)*, UPCR (g/g)^#^ | 7.41 * | - | - | - | - | - | - | - |
| UAE (mg/day)*, UACR (g/g)^#^ | 5.99 * | 4.23 ^#^ | 2.73 ^#^ | - | - | 0.88 ^#^ | 0.08 ^#^ | - |
| **Liver function** | | | | | | | | |
| Albumin (g/L) [35–52] | 28 | 33 | - | - | 28 | 31 | 32 | - |
| Bilirubin (mg/dL) [0.2–1.2] | 0.3 | - | - | - | 1.1 | 0.3 | - | 0.5 |
| AST (U/L) [<50] | 53 | - | - | - | 129 | - | 78 | 56 |
| ALT (U/L) [<50] | 60 | 54 | 38 | - | 143 | 76 | 55 | 48 |
| GGT (U/L) [<60] | 88 | 168 | 212 | - | 480 | 341 | 216 | 236 |
| ALP (U/L) [40–130] | 97 | 127 | 146 | - | 277 | 242 | - | 198 |
| INR [0.9–1.2] | 1.04 | - | - | - | 1.07 | 1.08 | - | 1.19 |
| AFP (ng/mL) [<7] | - | - | - | - | 1558 | 8129 | 42994 | - |
| **Additional parameters** | | | | | | | | |
| Na (mmol/L) [135–145] | 140 | 140 | 140 | - | 132 | 136 | 142 | 144 |
| LDL (mg/dL) [<116] | 158 | 104 | - | - | - | 63 | 58 | - |
| LDH (U/L) [<250] | 158 | 199 | 207 | - | 341 | 236 | - | 154 |

Abbreviations: AFP, alpha-fetoprotein; ALP, alkaline phosphatase; ALT, alanine aminotransferase; AST, aspartate aminotransferase; eGFR, estimated glomerular filtration rate (CKD-EPI, Chronic Kidney Disease Epidemiology Collaboration equation); GGT, gamma-glutamyl transferase; Hb, hemoglobin; INR, international normalized ratio; LDH, lactate dehydrogenase; LDL, low-density lipoprotein; MCV, mean corpuscular volume; Na, sodium; UAE, urinary albumin excretion; UACR, urinary albumin-to-creatinine ratio.
